# Illness Perceptions of Patients with Occupational Skin Diseases in a Healthcare Centre for Tertiary Prevention: A Cross-Sectional Study

**DOI:** 10.3390/ijerph20095652

**Published:** 2023-04-26

**Authors:** Marc Rocholl, Annika Wilke, Julia Meyer, Swen Malte John, Michaela Ludewig

**Affiliations:** 1 Institute for Health Research and Education, Department of Dermatology, Environmental Medicine and Health Theory, University of Osnabrück, Am Finkenhügel 7a, 49076 Osnabrück, Germany; 2 Institute for Interdisciplinary Dermatological Prevention and Rehabilitation (iDerm), University of Osnabrück, Am Finkenhügel 7a, 49076 Osnabrück, Germany

**Keywords:** contact dermatitis, eczema, illness perceptions, self-regulation model, cross-sectional studies, Germany

## Abstract

**Objectives:** To investigate the illness perceptions of patients with occupational skin diseases (OSDs). **Design:** Cross-sectional study. **Setting:** Specialised healthcare centre for inpatient and outpatient individual prevention in occupational dermatology in Germany. **Participants:** A total of 248 patients with hand eczema (55.2% female; average age: 48.5 years, SD: 11.9) were included in the final analyses. **Measures:** A modified and recently validated version of the ‘Revised Illness Perception Questionnaire’ (IPQ-R) was used to assess illness perceptions. Severity of skin disease was evaluated with the Patient-Oriented Eczema Measure (POEM), the Osnabrueck Hand Eczema Severity Index (OHSI), and a single, self-reported global item. The Erlangen Atopy Score (EAS) was used for atopy screening. **Results:** We found strong illness identity, high emotional impact, and long timeline beliefs, meaning that study participants perceive their OSD on the hands as a highly symptomatic, emotionally burdening, and chronic condition. Results suggest that hand eczema has a major impact on how participants manage their own lives, particularly during everyday life and occupational activities. Study participants predominantly identified irritant or sensitising substances and activities at work as well as skin protection regimes as causes of their disease. **Conclusions:** Healthcare workers should consider the illness perceptions as well as the disease burden of patients with an OSD on the hands in clinical practice. Multi-professional approaches to patient care should be sought. Illness perception in (occupational) dermatological patients should be the subject of further research.

## 1. Introduction

Occupational skin diseases (OSDs) are a very common condition in many European countries and account for up to 40% of all recognised occupational diseases [1]. The vast majority of OSDs—about 90% of all recognised OSD cases in Germany, for example—manifest as contact dermatitis (CD) on the hands [2,3]. Hairdressers [4,5,6], healthcare workers [7,8], construction workers [9,10], mechanics [11,12], and metal workers [13] are amongst the occupational groups at a particularly high risk of developing OSD. Aetiologically, OSD can be classified as irritant (also referred to as cumulative-subtoxic) contact dermatitis (ICD) or allergic contact dermatitis (ACD). ICD is the most common OSD and can result from direct occupational skin exposure to irritants, such as wet work, soap, solvents, and cutting fluids, or physical influences (e.g., pressure or friction), whereas ACD is a delayed-type immunological reaction and therefore results from exposure to a specific contact allergen (e.g., fragrance or preservative, epoxy resin, vulcanization accelerator) [3,14,15,16,17]. Moreover, OSDs often occur in combination with constitutional skin diseases such as atopic dermatitis (AD) or psoriasis. In this case, exogenous factors (irritants or allergens) can trigger and/or exacerbate the constitutional disease; AD, for example, is considered an important risk factor for the development of an occupational CD [3,15,17,18,19,20].

The clinical manifestations of ICD, ACD, and AD are sometimes rather similar and may therefore be difficult to distinguish. Typically, they are characterised by redness, scaling, itching, swelling, or cracking of the skin. Furthermore, overlapping or mixed diagnoses are frequent in clinical practice [2]. Unless the identification and, subsequently, elimination of the underlying cause(s) of ICD or ACD fail, CD may become a chronic condition [21]. Due to the potentially chronic and relapsing course, OSDs are associated with a high burden of disease, e.g., an impaired well-being, reduced quality of life, and higher levels of anxiety and depression [22,23,24,25,26,27]. Additionally, OSDs have a major socio-economic impact, e.g., through absence from work, job loss, early retirement or retraining, and high direct and indirect costs for society [28,29].

Against this background, primary and secondary prevention of OSD, as well as inpatient rehabilitation (tertiary prevention) in the case of severe OSD, which in Germany is predominantly referred to as ‘tertiary individual prevention’ (TIP), is of pivotal importance [30,31]. In various European countries, structured prevention programmes have been developed, evaluated, and implemented in standard care [32]. In Germany, in particular, secondary and tertiary individual prevention programmes have been long established within the framework of the so-called ‘dermatologist procedure’. In addition to regular outpatient dermatological diagnostics and treatment, interprofessional outpatient health education and counselling concepts (commonly called ‘skin protection seminars’) aiming at improving the individual skin protection and skin care behaviour are essential cornerstones of the procedure. In the case of severe OSD, when outpatient treatment is insufficient, or there is an unclear diagnosis or poor prognosis, three-week-inpatient treatment (TIP-programme) in specialised healthcare centres for tertiary prevention is indicated according to the dermatologist procedure [30,31,33,34,35,36]. 

Considering the high burden of disease for people with OSD, coping with the disease, defined as actively managing situations or events (e.g., health threats) that are perceived as psychologically and/or physically stressful, is particularly important [37,38,39]. In order to understand the mechanisms underlying the (predominantly unconscious) coping processes, it is crucial to investigate the patients’ perspective on their illness. These ‘illness perceptions’ (also called ‘illness representations’) are critical determinants of coping behaviour (e.g., treatment adherence). They are shaped by individual experiences and observations of the symptoms, the course of the disease, as well as social, cultural, and/or professional influences (e.g., common knowledge about treatment) [37,38]. Leventhal’s Common-Sense Model of Self-Regulation of Health and Illness (CSM) is a widely used and well-studied framework to describe and understand coping processes and to structure patients’ perspectives on illness [38,40]. A basic assumption of the CSM is that a perceived internal or external stimulus (e.g., a potential or experienced health threat, receiving a diagnosis) initiates self-regulation processes on a cognitive and emotional level. The CSM furthermore assumes that patients form unconscious emotional and cognitive representations which influence coping strategies. The chosen coping behaviours are evaluated in terms of their success or failure, which may lead to a modification of one’s own illness perceptions [41,42,43]. In the CSM, illness perceptions are organised in six different dimensions: *Identity* (given labels as well as perceived clinical signs and/or symptoms assigned to the disease); *Timeline* (duration and course of the disease, e.g., acute, chronic, cyclical); *Consequences* (expected and perceived social, economic, psychological, and physiological impacts); *Controllability* (perceived level of personal control and treatment control over a disease); *Causes* (one’s own beliefs about the cause(s) of the disease); and *Coherence* (extent to which the disease is comprehensive to an individual) [37,41]. 

In order to systematically assess these dimensions, various questionnaires have been developed: The ‘Revised Illness Perception Questionnaire’ (IPQ-R) [44], a revised version of the previously developed ‘Illness Perception Questionnaire’ (IPQ) [45], and the ‘Brief Illness Perception Questionnaire’ (B-IPQ) [46] are among the most frequently used instruments [43]. These have already been translated into a variety of languages as well as adapted and validated for several diseases [47,48]. A number of studies previously systematically investigated the illness perceptions of patients with skin diseases, e.g., psoriasis [49,50,51], AD [52,53], vitiligo [54], and alopecia areata [55], by using one of the above-mentioned questionnaires. In one of our previous works [56], we summarised the illness perceptions of patients with eczematous skin diseases, including (occupational) ICD and ACD, as well as AD. We found that studies measuring illness perceptions of patients with occupational ICD or ACD predominantly either use qualitative research methods, e.g., semi-structured interviews or focus groups [57,58,59] or the B-IPQ [60]. However, to the best of our knowledge, no study has examined the illness perceptions of patients with OSD of the hands using the IPQ-R. The aim of this cross-sectional study is therefore to investigate the illness perceptions of patients with OSD on the hands in a specialised healthcare centre for tertiary prevention in occupational dermatology in Germany and to describe how patients perceive their illness.

## 2. Methods

This study was approved by the ethics committee of the Osnabrück University (no. 13/2020; 8 April 2020). The report follows the STROBE (Strengthening the Reporting of Observational studies in Epidemiology) cross-sectional reporting guidelines [61]. A completed STROBE checklist is attached in Supplementary File S1. 

### 2.1. Design, Setting and Study Participants

We conducted a cross-sectional study in a specialised healthcare centre for inpatient and outpatient individual prevention in occupational dermatology in Osnabrück, Germany, from June 2020 to May 2021. Patients admitted to the three-week inpatient TIP-programme were invited to participate in the study at the end of the introductory seminar, which patients attend on the day of their admission. Participation in the study was voluntary. The participants of the seminar were informed that non-participation in the study or withdrawal of consent would not result in any disadvantages. Data were collected by means of a written questionnaire; therefore, sufficient German language skills were a prerequisite for study participation. Medical parameters (e.g., diagnosis, atopic disposition) were assessed by the attending dermatologist for each patient on day 1 of the TIP-programme. Inclusion criteria were the existence of hand eczema, age ≥ 18 years, and written informed consent. Patients who were exclusively diagnosed with occupational airborne contact dermatitis, occupational foot eczema, or tinea manuum, but did not have hand eczema currently or in the past, were excluded from the study. Study recruitment was predominantly undertaken by the first author.

The questionnaire applied in the study was pilot tested with seven patients admitted to the TIP-programme. The patients were instructed, for example, to evaluate the comprehensibility of the questions. Furthermore, the time required to complete the questionnaire was measured. Subsequent to the pilot test, the questionnaire was adapted based on patient feedback. Beyond this, patients or the public were not involved in the planning, conduct, or interpretation of the study.

### 2.2. Data Collection and Assessment Instruments

Data were collected through a self-assessed written questionnaire. We obtained sociodemographic data (e.g., age, sex, family status, smoking status, education, occupation, and employment status) first, followed by illness perceptions.

### 2.3. Illness Perceptions 

The primary outcome of this study (illness perceptions) was assessed using the German version of the IPQ-R [62]. The questionnaire was modified and adapted to patients with OSD on the hands. For this purpose, the term ‘hand eczema’ was used instead of the term ‘illness’. The modifications are based on the results of Rocholl et al. [56] and are described in detail by Ludewig et al. [63]. In short, the modified questionnaire consists of three parts with a total of 85 items. The first part of the IPQ-R is a symptom checklist that uses a sum score to measure experienced illness identity based on a yes–no scale. In addition to the 14 symptoms of the original IPQ-R [44,62], 12 specific signs and/or symptoms of hand eczema (e.g., ‘*redness*’, ‘*itch*’, ‘*dryness*’) were added. The other dimensions, except for the causes, were examined using 27 original and 4 additional items on perceived or expected consequences [63]. The items are rated on a five-point Likert scale (‘*strongly disagree*’ to ‘*strongly agree*’). High values represent stronger perceptions of illness. The IPQ-R concludes with 16 of the 18 original causes amended by 14 additional hand eczema-specific causes (e.g., ‘*hormonal fluctuations*’, ‘*hand washing*’, ‘*wearing gloves*’). These are also rated using a 5-point Likert scale. The modified IPQ-R was evaluated regarding its psychometric properties [63]. Although the original factorial structure of the IPQ-R described by Moss-Morris et al. [44] could not be replicated, inter alia because the factor ‘cyclical timeline’ could not be reproduced, Ludewig et al. [63] found a 7-factorial model with 29 items to assess illness perceptions and a solution consisting of 8 factors and 30 items for causes for the OSD-specific IPQ-R with overall satisfactory psychometric properties.

### 2.4. Diagnosis

The diagnosis of the skin disease was assessed by the patient’s attending dermatologist on day 1 of the TIP-programme. Since mixed diagnoses (e.g., ICD and AD) are common in this cohort [2], a tick box template was used in which multiple answers were possible for diagnosis of the skin disease on the hands. 

As mentioned above, AD is also considered an important risk factor for occupational CD. Therefore, the Erlangen Atopy Score (EAS) [64,65] was applied to evaluate the atopic disposition of the study participants. The EAS assesses the likelihood of an atopic diathesis based on a total of 24 anamnestic and clinical features, e.g., family anamnesis, self-anamnesis, atopic stigmata, or laboratory values. Between 0.5 and 3 points can be given per feature. In total, the maximum score is 42. The values are interpreted as follows: 0–3 ‘*no atopic skin diathesis*’; 4–7 ‘*improbable atopic skin diathesis*’; 8–9 ‘*indistinct atopic skin diathesis*’; >10 ‘*atopic skin diathesis*’.

### 2.5. Disease Severity

The severity of the skin disease was assessed in three different ways. Self-reported severity was measured in accordance with Coenraads et al. [66] using a global item (‘*How would you describe the severity of your hand eczema?*’) with a five-point scale: (1) *clear*; (2) *almost clear*; (3) *mild*; (4) *moderate*; (5) *severe*. In this context, we additionally asked the participants about the duration of their skin disease.

Furthermore, the Patient-Oriented Eczema Measure (POEM) [67,68] was used. The POEM is a short, easy, and quick-to-use questionnaire developed to measure the disease severity from the perspective of patients with AD. The instrument was developed with patient involvement and showed good psychometric properties (e.g., in terms of internal consistency; Cronbach’s Alpha: 0.88) [68]. Its use in clinical trials is recommended by the Harmonising Outcome Measures for Eczema (HOME) initiative [69]. The POEM assesses disease severity by asking about the frequency of symptoms during the last 7 days on a 5-point scale: (0) *No Days*; (1) *1–2 Days*; (2) *3–4 Days*; (3) *5–6 Days*; (4) *Every Day*. Since the POEM was developed for patients with AD, we replaced the term ‘*eczema*’ with the disease-specific pattern ‘*hand eczema*’, which is relevant for our study. Due to the one-factor structure of the POEM, a maximum score of 28 points can be achieved. Higher scores indicate a higher disease severity. In detail, the PEOM assessments can be interpreted as follows: 0–2 *Clear or almost clear*; 3–7 *Mild hand eczema*; 8–16 *Moderate hand eczema*; 17–24 *Severe hand eczema*; 25–28 *Very severe hand eczema*. Although the POEM was developed for patients with AD, we decided to use it, in line with the recommendations of the HOME initiative. 

Finally, a clinical assessment of objective disease severity was carried out on day 1 of the TIP-programme by the attending dermatologist using the Osnabrueck Hand Eczema Severity Index (OHSI) [70], an easy-to-use, simple, and practicable tool used in various clinical and epidemiological studies [2,33,34,71,72,73]. The OHSI evaluates the disease severity through a set of six characteristics (namely erythema, scaling, papules, vesicles, infiltration, and fissures). OHSI scores can range from 0 points to a maximum of 18 points, with higher scores representing higher disease severity [70,74].

### 2.6. Statistical Analyses

Statistical analyses were carried out using IBM SPSS 28 [75]. The data input was controlled randomly. A total of 34.7% (N = 88) of all data sets were checked for errors during data input. A completely verified data set of one questionnaire contained 246 items (average error rate: 0.6% per data input). Descriptive statistics were calculated for all variables. The mean (M), median (Md), and standard deviation (SD) were calculated for continuous variables (e.g., age, duration of disease, IPQ-R). Categorial data (e.g., sex, family status) are presented as frequencies and percentages. In the case of missing values caused by participants omitting single questions, these are reported in the tables as the differences required to sum to 100% (‘*not specified/prefer not to say*’). Percentage scores for illness beliefs were calculated by summing scores for ‘*agreeing*’ and ‘*strongly agreeing*’ with each item.

## 3. Results

### 3.1. Participants

During the survey period, 291 patients took part in the introductory seminar on the first day of the TIP-programme. Of these, 254 (87.3%) gave informed consent to participate in the study. Subsequent to data collection, six participants were excluded: Two patients were excluded due to a large number of missing values as a result of a language barrier and another four patients because the medical examination did not reveal any history or presence of hand eczema, resulting in 248 participants being included in the final analysis. Sociodemographic characteristics of patients enrolled in the study are shown in Table 1.

Table 2 shows the severity of the OSD of the hands assessed with the OHSI, the POEM, and the global self-assessment. It also presents the results of atopy screening. Most patients reported moderate to severe hand eczema, assessed with the POEM (75.5%; N = 187) and the global self-assessment (78.7%; N = 195). Atopy screening revealed that 41.5% (N = 103) of the study participants have an atopic diathesis according to EAS. Moreover, the data analysis found 166 study participants (66.9%) to have skin lesions on other parts of the body (e.g., feet or face) in addition to their hand eczema.

Results of the medical examination of the study participants, especially with regard to the classification of their hand eczema, are shown in Table 3. Overlapping diagnoses, i.e., more than one subtype of hand eczema (e.g., atopic and irritant hand eczema), were observed in 147 participants (59.3%, N = 248).

### 3.2. Ilness Identity

**Figure 1:**
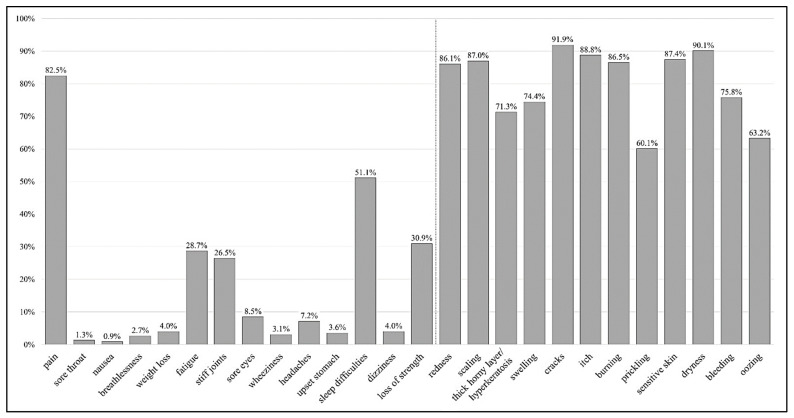
Percentage of participants believing certain clinical signs and/or symptoms are related to their hand eczema (N = 223, multiple responses possible). **Figure legend:** The bars show the percentage of study participants attributing perceived clinical signs or symptoms to their hand eczema. The first 14 items (‘*pain*’ until ‘*loss of strength*’) represent the original identity scale of the IPQ-R. The additional 12 items are specific clinical signs and/or symptoms of hand eczema added for the purpose of this study.

Assessment of illness identity revealed ‘*cracks*’ (91.9%), ‘*dryness*’ (90.1%), and ‘*itch*’ (88.8%) as the most common clinical signs or symptoms assigned to hand eczema (see Figure 1). More than half of the study participants (51.1%) reported ‘*sleep difficulties*’ due to their hand eczema. Furthermore, we found that of the ten most frequently mentioned clinical signs and/or symptoms, only one item (‘*pain*’) belongs to the original IPQ-R identity scale. Percentages of study participants attributing perceived clinical signs or symptoms to their hand eczema are shown in detail in Table S1 (see Supplementary File S2). On average, each study participant attributed more than 12 symptoms (M = 12.2, SD = 3.9; N = 223) to his or her hand eczema.

### 3.3. Illness Perceptions

The mean scores of the IPQ-R subscales are provided in Table 4. We found high scores on the timeline subscale, implying that study participants perceive their hand eczema as a long-lasting, chronic condition. The following items, which are included in the subscale ‘timeline’, highlight this point: ‘*My hand eczema will last a short time*’ (IP_1: 88.9% disagree/strongly disagree; N = 235); ‘*My hand eczema will last for a long time*’ (IP_2: 83.9% agree/strongly agree; N = 242); and ‘*My hand eczema will pass quickly*’ (IP_23: 80.0% disagree/strongly disagree; N = 231).

Study participants additionally claimed that their OSD on the hands has a major impact on their own lives. The original IPQ-R items IP_5 (‘*My hand eczema has major consequences on my life*’; agree/strongly agree: 85.1%; N = 242) as well as the negatively worded item IP_6 (‘*My hand eczema does not have much effect on my life*’; disagree/strongly disagree: 79.6%; N = 240) indicate that the OSD on the hands has serious consequences. Furthermore, the added items N_2 (‘*My hand eczema has an impact on activities of daily life (e.g., cleaning, washing dishes, etc.)*’; agree/strongly agree: 93.0%; N = 245), N_1 (‘*My hand eczema has an impact on my professional activity*’; agree/strongly agree: 91.3%; N = 242), and, somewhat less pronounced, N_3 (‘*My hand eczema has an impact on my leisure activities (e.g., hobbies, social activities*’; agree/strongly agree: 78.7%; N = 244), confirm these findings. 

In addition to these findings, study participants reported negative emotional effects related to their OSD on the hands: Two-thirds of the study participants reported feelings of depression because of the hand eczema (IP_28: ‘*I get depressed when I think about my hand eczema*’; agree/strongly agree: 66.7%; N = 240). More than half of the participants reported being worried (IP_29: ‘*When I think about my hand eczema I get upset*’; agree/strongly agree: 56.0%; N = 243) or angry (IP_30: ‘*My hand eczema makes me feel angry*’; agree/strongly agree: 52.3%; N = 243) because of the hand eczema.

### 3.4. Illness Causes

Descriptive statistics for the eight cause subscales of the modified IPQ-R are shown in Table 4. Study participants described ‘*skin-irritating substances at work*’ (NC_4: 89.4% agree/strongly agree; N = 246), ‘*activities at work*’ (NC_2: 88.6% agree/strongly agree; N = 246), and ‘*sensitising substances at work*’ (NC_6: 74.2% agree/strongly agree; N = 244) as the most important causes of hand eczema. Besides ‘*hand disinfection*’ (NC_9: 70.1% agree/strongly agree; N = 244) and ‘*hand washing*’ (NC_8: 61.7% agree/strongly agree; N = 246), ‘*wearing gloves*’ (NC_11: 58.6% agree/strongly agree; N = 244) is perceived as an important workplace-related cause for hand eczema by more than half of the study participants. Among the psychological causes, ‘*stress or worry*’ (C_1: 62.5% agree/strongly agree; N = 243) is the most frequently mentioned cause by the study participants. For most of the other items of the cause subscale, the values vary—more or less markedly—around the middle response category ‘*neither agree nor disagree*’.

## 4. Discussion

The aim of this cross-sectional study was to investigate the illness perceptions of patients with OSD on the hands and to describe how these patients perceive their illness. For this purpose, we conducted a questionnaire survey in a specialised healthcare centre for tertiary prevention in occupational dermatology in Germany using a modified version of the IPQ-R. We found strong illness identity, high emotional impact, and long timeline beliefs in our sample, meaning that study participants perceive their hand eczema as a highly symptomatic, emotionally burdening, and chronic condition. Furthermore, our results suggest that hand eczema has major impact on how participants manage their own lives, particularly during everyday life and occupational activities. Finally, it was observed that study participants predominantly identified irritant or sensitising substances and activities at work, as well as skin protection and skin care regimes (e.g., hand disinfection, hand washing, wearing gloves) as causes of their hand eczema.

Our study shows that itching is one of the three most frequently mentioned symptoms attributed to hand eczema. Yet, itching is an often-underestimated symptom of various skin diseases of the hands [76] with a significant impact on the affected individuals. Wittkowski et al. [53], for example, describe a sample of AD patients, of whom 98.6% experienced itching due to their AD. Furthermore, the authors observed that about two-thirds (66.2%) of the study participants (N = 284) described sleep difficulties and more than one-third (36.3%) described fatigue in relation to their AD [53]. Similar results are also found in our sample, which suggests that sleep difficulties and fatigue are frequently attributed to an eczematous skin disease, especially an OSD in the hands. Overall, the strong illness identity we found in our study and the experience of the hand eczema as highly symptomatic is equally described in patient groups with occupational and non-occupational CD [56]. Meta-analyses of the IPQ and the IPQ-R aiming at investigating interrelationships between the dimensions of illness perceptions and illness outcomes or coping behaviours, respectively, furthermore showed that a strong illness identity is associated with various illness outcomes (e.g., negatively associated with psychological well-being, vitality, role, and social functioning; positively associated with psychological distress) and coping behaviours (e.g., positively associated with the coping behaviours ‘avoidance/denial’ and ‘emotion expression’) [47,77]. Broadbent et al. [48] derived similar conclusions in their systematic review and meta-analysis of the B-IPQ: They observed a strong illness identity being associated with more depression and anxiety. Higher levels of anxiety and depression have in turn also been described for patients with (occupational) CD on the hands [22,23,27]. Consistent with these findings, we also found negative emotional effects of OSD on the hands, in particular in terms of depression, worry, and anxiety, although it is important to note that we did not assess depression or anxiety with a separate standardised questionnaire (e.g., the Hospital Anxiety and Depression Scale [78]). To sum up, it can be assumed that OSD on the hands may result in a considerable somatic and psychological burden. To ensure adequate and targeted treatment of patients in practice, the high burden of disease associated with OSD of the hands needs to be considered by healthcare workers.

From a methodological point of view, it is important to mention that nine of the ten most frequently mentioned symptoms or signs of the identity subscale in our study were newly added when adapting the questionnaire to OSD. This, on the one hand, highlights the importance of disease-specific adaptation of the IPQ-R in future studies, in particular the identity subscale. However, on the other hand, it is also important to note that some study participants apparently had problems understanding the presentation format of the subscale, as 19 participants (7.7%; N = 248) did not provide information about the identity of their hand eczema. The way the subscale was presented could therefore influence the results of this study. In future studies it may be considered either to provide more detailed instructions on how to complete the subscale or to redesign the way the items are presented.

In terms of disease severity, most of our study participants reported to have moderate to severe hand eczema. The clinical assessment of hand eczema severity using the OHSI provided similar results compared to previous studies in the same setting [2,33]. Cohort studies in outpatient settings, in comparison, report slightly lower OHSI scores compared to our findings [34,35]. Our analysis revealed that study participants consider their professional activities and the substances they are exposed to as the most important causes of their skin disease. In view of our study sample, a primarily occupational cause attribution is hardly surprising: Since all patients admitted to the TIP-programme receive care within the ‘dermatologist procedure’, it is likely that this influences their illness perceptions, in particular in view of the perceived causes of their hand eczema. However, we observed overlapping or mixed diagnoses in almost 60% of the participants, which has also been described in similar study populations [2]. Therefore, it is possible that the subjects in our study overestimated the influence of professional activities and exposures on their skin disease while underestimating the influence of an endogenous influence (e.g., an atopic skin diathesis). In one of our previous works [56], we summarised suspected causes of patients with occupational and non-occupational eczematous skin diseases and found a higher level of heterogeneity in terms of perceived causes, namely, endogenous causes (e.g., psychological factors) and exogenous causes (e.g., behavioural, environmental, and occupational factors). At this point, however, it should be noted that different data collection methods were used, which may have influenced these results. Nevertheless, perceived causes have a major impact on how people cope with a disease (e.g., seeking and adhering to treatment, changing health behaviour), so it is crucial for healthcare workers to elaborate and discuss with patients their perceived causes of their illness [37].

### Strengths and Limitations of This Study

This study has a number of strengths and limitations. The cross-sectional design of our study does not permit any conclusions regarding causality. Moreover, despite the application of a modified and validated version of the IPQ-R, which is a major strength of this study, we cannot provide information on all dimensions of the CSM (e.g., regarding the dimension ‘cyclical timeline’) or on the sensitivity to change in our questionnaire (e.g., in the context of intervention studies). Therefore, future studies should inter alia examine sensitivity to change more closely using a longitudinal study design. When planning longitudinal studies, indeed, it should be considered that filling out a long questionnaire—such as the modified IPQ-R used in our study—is associated with a considerable effort for patients. For that reason, the use of the B-IPQ may also be considered if necessary. Although the B-IPQ is less detailed compared to the IPQ-R, the use of the B-IPQ requires less time and effort. Interpretation of the B-IPQ is also straightforward, which is particularly important in clinical settings where data collection and data analysis must be quick, and in longitudinal studies with follow-up assessments as well [46,48].

Furthermore, the transferability of our results to other patient cohorts or person groups is limited: Firstly, the generalisability of the results to patients from other countries is limited due to the specific healthcare arrangements of the statutory accident insurance in Germany. Secondly, it should be noted that our study sample is very heterogeneous, e.g., in terms of diagnoses or socio-demographic characteristics, which makes it at least difficult to compare the results with those studies with more homogeneous cohorts (e.g., patients with AD). Finally, we conducted our study with patients who were admitted to a specialised healthcare centre for tertiary prevention in occupational dermatology to participate in an inpatient rehabilitation programme, and who therefore cannot be considered representative of all patients with an OSD on the hands in Germany, e.g., in terms of severity of the skin disease. There is no doubt, however, that our data represent findings in the high-risk population for contact dermatitis. Future studies could consider assessing the illness perception of patients with OSD in outpatient settings (e.g., skin protection seminars or dermatology practices). 

The CSM is a valuable framework for describing and understanding coping behaviours. As mentioned earlier, these can—depending on the individual, the context, or the stage of the person’s disease—occasionally be classified as adaptive and maladaptive coping strategies [47]. However, for the systematic assessment and classification of coping strategies and their effects, it is necessary to use standardised instruments (e.g., the COPE inventory [79]). Therefore, it could be considered a limitation of the present study that we did not use a standardised coping scale in our study. Future studies should thus use an instrument to categorise coping behaviours in order to examine the association between illness perceptions and coping behaviours.

Finally, although the overall size of our study sample is rather small, it can be stated that the high response rate of our study can, at the same time, be considered a strength. The major strength of this study, meanwhile, is that, to the best of our knowledge, it is the first time the illness perceptions of patients with OSD on the hands were investigated in a specialised healthcare centre for tertiary prevention in occupational dermatology using the commonly accepted and validated IPQ-R. 

## 5. Conclusions

In conclusion, patients with an OSD of the hands seem to experience their skin condition as a significant burden. This is reflected in various dimensions of the CSM. For healthcare workers, it is particularly important to consider this burden in clinical practice. Given the complexity of illness perceptions, as well as the impact of OSD on the hands, a multi-professional approach to patient care should ideally be pursued.

## Figures and Tables

**Table 1:** Characteristics of study participants (N = 248).

	Frequency (N)	Percentage (%)
**Sex**		
female	137	55.2%
male	111	44.8%
**Age**		
M (SD)	48.5 years (11.9)	
range	18–66	
18–20 years	3	1.2%
20–29 years	28	11.3%
30–39 years	26	10.5%
40–49 years	35	14.1%
50–59 years	118	47.6%
60–69 years	38	15.3%
**Education**		
no school-leaving qualification	1	0.4%
secondary school/elementary school leaving certificate	71	28.6%
intermediate school-leaving certificate/secondary school leaving certificate	112	45.2%
advanced technical college certificate	29	11.7%
general higher education entrance qualification/A-levels	19	7.7%
bachelor’s degree	3	1.2%
master’s degree/diploma	5	2.0%
doctoral degree	2	0.8%
not specified/prefer not to say	6	2.4%
**Marriage**		
yes	186	75.0%
no	60	24.2%
not specified/prefer not to say	2	0.8%
**Smoking status**		
yes	92	37.1%
no	148	61.7%
not specified/prefer not to say	8	3.2%
**Duration of disease** (N = 234)		
M	97.6 months ≙ 8.1 years	
SD	126.7 months ≙ 10.6 years	
Md	48.0 months ≙ 4.0 years	
min	2.0 months ≙ 0.2 years	
max	720.0 months ≙ 60.0 years	
**Occupation groups**		
healthcare/elderly care	87	35.1%
metal workers	10	4.0%
mechanics	36	14.5%
hairdressers, cosmetologists, close contact services	8	3.2%
construction workers	12	4.8%
food handlers	18	7.3%
cleaning personal	2	0.8%
gardeners/florists	3	1.2%
others	60	24.2%
not specified/prefer not to say	12	4.8%
**Employment**		
self-employed	6	2.4%
employed	237	95.6%
unemployed	4	1.6%
not specified/prefer not to say	1	0.4%

M = mean; max = maximum; Md = median; min = minimum; SD = standard deviation.

**Table 2:** Severity of OSD and results of atopy screening (N = 248).

	Frequency (N)	Percentage (%)
**OHSI** (N = 247)		
M (SD)	6.6 (3.1)	
range	0–15	
**POEM** (N = 241)		
M (SD)	14.4 (6.6)	
Md	15.0	
range	0–28	
0–2 (clear or almost clear)	9	3.6%
3–7 (mild *hand* eczema)	30	12.1%
8–16 (moderate *hand* eczema)	110	44.4%
17–24 (severe *hand* eczema)	77	31.1%
25–28 (very severe *hand* eczema)	15	6.1%
excluded due to 2 or more missing values	7	2.8%
**Global self-assessment**		
clear	3	1.2%
almost clear	11	4.4%
mild	26	10.5%
moderate	145	58.5%
severe	50	20.2%
not specified/prefer not to say	13	5.2%
**Erlangen Atopy Score** (N = 247)		
M (SD)	8.7 (5.0)	
Md	8.0	
range	0–24	
0–3 (no atopic skin diathesis)	39	15.7%
4–7 (improbable atopic skin diathesis)	75	30.2%
8–9 (indistinct atopic skin diathesis)	31	12.5%
>10 (atopic skin diathesis)	103	41.5%

EAS = Erlangen Atopy Score; M = mean; Md = median; OHSI = Osnabrueck Hand Eczema Severity Index; POEM = Patient-Oriented Eczema Measure; SD = standard deviation.

**Table 3:** Specifying the diagnosis of hand eczema (N = 241, multiple responses possible).

Diagnosis [Hands]	Frequency	Percentage of All Responses (N = 406)	Percentage of All Cases (N = 241)
atopic hand eczema	160	39.4%	71.4%
irritant hand eczema	160	39.4%	71.4%
allergic hand eczema	41	10.1%	18.3%
hyperkeratotic-rhagadiform hand eczema	42	10.3%	18.8%
not-classifiable hand eczema	3	0.7%	1.3%

**Table 4:** Descriptive statistics of the modified IPQ-R subscales [63].

	IPQ-R-Subscales	Possible Scores	N	Min–Max	Md	M (SD)
*Illness identity (26 items)*						
1.	illness identity	0.0–26.0	223	1.0–23.0	12.0	12.2 (3.9)
*Perceptions about illness (29 items)*						
1.	timeline	3.0–15.0	239	3.0–15.0	12.0	12.2 (2.0)
2.	consequences: impact on one’s own life	5.0–25.0	247	7.0–25.0	21.0	20.8 (3.4)
3.	consequences: financial and social impact	4.0–20.0	245	4.0–20.0	12.0	11.8 (3.6)
4.	personal control	4.0–20.0	244	4.0–20.0	12.0	12.1 (3.3)
5.	treatment control	3.0–15.0	240	3.0–15.0	11.0	10.5 (2.2)
6.	coherence	5.0–25.0	243	5.0–25.0	14.0	14.8 (4.6)
7.	emotional representations	5.0–25.0	244	5.0–25.0	17.0	16.5 (4.6)
*Causes (30 items)*						
1.	psychological causes	7.0–35.0	245	7.0–32.0	18.0	18.2 (5.3)
2.	causes occurring outside of work	4.0–20.0	245	4.0–20.0	12.0	11.9 (3.5)
3.	skin protection regime	3.0–15.0	247	3.0–15.0	11.0	10.7 (2.7)
4.	behavioural risk factors	3.0–15.0	245	3.0–15.0	6.0	6.0 (2.3)
5.	immunity	4.0–20.0	244	4.0–20.0	11.0	10.9 (3.1)
6.	causes at work	3.0–15.0	247	3.0–15.0	12.0	12.1 (2.1)
7.	other risk factors	4.0–20.0	244	4.0–20.0	10.0	10.5 (2.8)
8.	climatic conditions	2.0–10.0	243	2.0–10.0	6.0	6.0 (2.1)

M = mean; max = maximum; Md = median; min = minimum; SD = standard deviation.

## Data Availability

The datasets used and analysed during the current study are available from the corresponding author on reasonable request.

## Supplementary Materials

The following supporting information can be downloaded at: https://www.mdpi.com/article/10.3390/ijerph20095652/s1, Supplementary File S1: Completed STROBE checklist; Table S1: Percentage of participants attributing clinical signs and/or symptoms to their hand eczema (N = 223, multiple responses possible).

## Abbreviations

ACD	allergic contact dermatitis
AD	atopic dermatitis
B-IPQ	Brief Illness Perception Questionnaire
CD	contact dermatitis
CSM	Common-Sense Model of Self-Regulation of Health and Illness
EAS	Erlangen Atopy Score
HOME	Harmonising Outcome Measures for Eczema
ICD	irritant contact dermatitis
IPQ	Illness Perception Questionnaire
IPQ-R	Revised Illness Perception Questionnaire
M	mean
Max	maximum
Md	Median
Min	minimum
OHSI	Osnabrueck hand eczema severity index
OSD	occupational skin diseases
POEM	Patient-Oriented Eczema Measure
SD	standard deviation
STROBE	Strengthening the Reporting of Observational studies in Epidemiology
TIP	tertiary individual prevention
