# Supplementary material for: Illness Perceptions of Patients with Occupational Skin Diseases in a Healthcare Centre for Tertiary Prevention: A Cross-Sectional Study
Source: Int J Environ Res Public Health. 2023 Apr 26;20(9):5652. doi: 10.3390/ijerph20095652 (PMC10178866; doi:10.3390/ijerph20095652)
Supplement: Supplementary file 1 [file ijerph-20-05652-s001.zip › Supplementary File S2.pdf]

ADDITIONAL FILE S2

Additional file S2: Table S1.

Table S1

Table S1: Percentage of participants attributing clinical signs and/or symptoms to their hand eczema (N = 223, multiple responses possible).

	symptoms related to hand eczema	frequency	percentage of all responses N = 2.716
1.	pain	184	6.8%
2.	sore throat	3	0.1%
3.	nausea	2	0.1%
4.	breathlessness	6	0.2%
5.	weight loss	9	0.3%
6.	fatigue	64	2.4%
7.	stiff joints	59	2.2%
8.	sore eyes	19	0.7%
9.	wheeziness	7	0.3%
10.	headaches	16	0.6%
11.	upset stomach	8	0.3%
12.	sleep difficulties	114	4.2%
13.	dizziness	9	0.3%
14.	loss of strength	69	2.5%
15.	redness	192	7.1%
16.	scaling	194	7.1%
17.	thick horny layer/hyperkeratosis	159	5.9%
18.	swelling	166	6.1%
19.	cracks	205	7.5%
20.	itch	198	7.3%
21.	burning	193	7.1%
22.	prickling	134	4.9%
23.	sensitive skin	195	7.2%
24.	dryness	201	7.4%
25.	bleeding	169	6.2%
26.	oozing	141	5.2%
